# Appendicitis: Its Special Features in Children

**Published:** 1907-03-02

**Authors:** J. P. Lockhart Mummery

**Affiliations:** Surgeon King Edward VII. Hospital, and Assistant Surgeon to the North-Eastern Hospital for Children and St. Mark's Hospital for Fistula, etc.


### Hospital Clinics.

### APPENDICITIS: ITS SPECIAL FEATURES IN CHILDREN.

By J. P. LOCKHART MUMMERY, F.R.C.S.Eng., Surgeon King Edward VII. Hospital, and Assistant Surgeon to the North-Eastern Hospital for Children and St. Mark's Hospital for Fistula, etc.

Lecture delivered at the Polyclinic, January 1907.

Appendicitis in children presents several important points, both in diagnosis and treatment, in which it differs from the same disease in adults. It is very uncommon under two years of age. Only a very few cases have been recorded of children who have been operated on for appendicitis at 20 months and 22 months. It is much more common after six years of age, and in Paris it appears to be more frequently met with in children than in adults. In London adult appendicitis is the more common.

The diagnosis of appendicitis in children is difficult, as children often have curious and misleading symptoms, and there is much greater variation in the symptoms than we find when appendicitis occurs in adults. It tends to run a much more rapid and acute course, the gangrenous form is much more common, and general peritonitis is a more frequent complication. The whole inflammatory process is more rapid in children, and there is not the same tendency to the formation of adhesions and to localisation of the products of inflammation that we are accustomed to see in adults.

### The Diagnosis.

We are obliged in children to depend very much less upon the patient's history and the account of the attack, and in some cases little or nothing can be learned. Gastric and abdominal pain, sickness, and diarrhœa are comparatively common affections of childhood, and it is not possible to get any reliable account of symptoms which point to appendicitis. Many people seem to think that " stomach-ache " is natural to children, and no special attention is paid to it.

Most children when ill are suspicious and intolerant of strangers, and resent examination unless it is most tactfully carried out. It is advisable, therefore, first of all, to ascertain from the mother or relations as much as possible of the child's history and the account of the present illness, and not to start by asking the child questions. It is important to observe the position in which the child lies. Notice whether the right thigh is drawn up, and, if so, gently straighten it and see if the child resents this and if it is again immediately flexed. It is best in a case of suspected appendicitis to proceed at once to examine the abdomen, as this is the important part of the examination, and it is advisable to investigate this before the child is tired of being examined. We may observe whether the abdominal wall moves with respiration or not, and may notice its general appearance. One of the most important points is to observe whether there is rigidity of the right rectus and other abdominal muscles. Rigidity of the muscles is a most important sign of intra-abdominal mischief, and I think ranks first in this particular.

Tenderness over the cæcum is also important, and we should observe whether on gentle pressure in this neighbourhood the abdominal muscles attempt to protect this part, and this observation should be compared with the facts of the opposite side. Of course if there is a definite tumour to be felt this assists the diagnosis very considerably. In a case I attended recently, however, this proved misleading.

The patient was a child, aged seven, who was sent to the hospital as a case of appendix abscess. On examination there was rigidity on the right side of the abdomen, and a large round tumour, which was very tender, could be felt on the right iliac fossa. There was a history of sickness and constipation, and of sudden onset of pain; the temperature was raised and the pulse was quick. Everything pointed to the diagnosis being correct, and I decided to operate. On opening the abdomen over the swelling, instead of finding an abscess, as I had expected, I found a large loop of semi-gangrenous bowel which proved to be a volvulus of the small intestine just above the ileo-cæcal valve.

Distension of the bowel is as a rule an important indication of a serious lesion, and shows that the condition has already reached an advanced stage. It often means that general peritonitis is present. The absence of distention, however, means little. The temperature is not of much importance, and personally I attach much more importance to the pulse. A quick pulse is, I believe, of the greatest importance as a sign of serious illness in children.

In children evidence of the greatest value can often be obtained by a rectal examination, and this important means of making a diagnosis should never be neglected. Many people, I think, do not seem to realise how large a part of the abdominal cavity in children can be felt by a finger in the rectum. Even a small baby's rectum will easily accommodate an ordinary sized man's finger without any fear of damage; and the whole of the pelvic organs, and in young children even the kidneys, can be felt quite easily through the walls of the sigmoid flexure. If necessary a little anæsthetic may be given, and in this way a thickened appendix or an abscess can often be felt when it could not be made out through the rigid abdominal wall. Lastly, in a difficult case one can often clear up the diagnosis by sitting quietly by the child's bedside for some time and watching it. One often gets a much clearer idea of the real condition of affairs in this way than is possible in a single examination.

As a rule the diagnosis of acute appendicitis in a child is not a difficult matter, but there are certain conditions in which there is extreme difficulty experienced in arriving at a correct conclusion, and in some cases it is quite impossible. Perhaps the most difficult cases are those where there is pneumonia with abdominal symptoms. The onset of the attack and the symptoms may closely resemble acute appendicitis or even peritonitis. In doubtful cases the chest should always be carefully examined for physical signs; if the physical signs of pneumonia are discovered the diagnosis is not difficult. The greatest difficulty, however, arises in those cases where the pneumonic process commences in the centre or near the base of the lung, and in which there is diaphragmatic pleurisy. In such cases it may be impossible to make a correct diagnosis until some days after the onset of the attack. Intestinal worms may also cause symptoms which closely resemble acute appendicitis; there may be fever and vomiting, and the diagnosis is only cleared up by the discovery of the worms in the evacuations. In typhoid with a sudden onset the diagnosis may also be difficult.

Considerable difficulty arises in those cases in which the child has symptoms of an acute peritonitis. Tubercular peritonitis with an acute onset, or which has not been diagnosed before acute symptoms have shown themselves, may closely resemble appendicitis. A rectal examination is here often of value. Fortunately a mistake in diagnosis is not of any serious import, as beneficial results usually follow laparotomy in these cases. Pneumococcic and gonococcic peritonitis may also give rise to difficulties, and an operation in these cases will do harm. Gonococcic peritonitis occurs exclusively in female children, and is a complication of vulvo-vaginitis, the infection having spread up the tubes. A rectal examination will usually enable these cases to be diagnosed, as the tube can be felt to be thickened and inflamed; the presence of vulvitis is also significant.

Chronic appendicitis in children is probably much commoner than is generally supposed. It is generally overlooked and the symptoms attributed to other causes. Many of the acute intestinal disorders of children which are accompanied by a rise in temperature, a general febrile disturbance, and abdominal pain and tenderness are subacute attacks of appendicitis. Often these attacks occur at frequent intervals, and the mother attributes them to the fact that the child has delicate bowels or a weak stomach and treats them herself without reference to a doctor. The attack usually subsides in a day or two; only, however, to recur again from some insignificant cause, until ultimately a more severe attack than usual occurs and an abscess forms. I think that we should always think of appendicitis when we see a child who has frequent attacks of colic accompanied by febrile symptoms. I have seen several cases of children who have been sickly and delicate with frequent alimentary troubles, and who, after the appendix has been removed, became strong, healthy children.

### Indications for Operations.

It is now, I think, agreed that in all cases of appendicitis in children, when a definite diagnosis has been made, an operation should sooner or later be performed for the removal of the appendix. There is still considerable difference of opinion as to whether the operation should be done at once or delayed until the acute attack has passed off. French surgeons advocate that, except in those cases when the operation can be performed at the very commencement of the attack, we should, whenever possible, wait till the acute symptoms have passed off. This seems at first very good advice, but the whole question resolves itself into one of diagnosis. If we can be certain that there is not a localised abscess which may, if left alone, burst into the peritoneal cavity, or a gangrenous appendix which, if not immediately removed, must quickly prove fatal, by all means let us wait. It is generally not very difficult to tell whether there is a localised abscess, but unfortunately it is often impossible to be certain that there is not a gangrenous appendix. Unfortunately, also, the proportion of acute gangrenous cases is larger in children than in adults, and the diagnosis is more difficult. We so frequently see cases in which a gangrenous appendix has not been operated upon early enough that I think we must be very careful before we accept this axiom in practice, though we may agree with it in theory.

No absolute rule can be made, and each case must, within certain limits, be treated upon its merits. If all the local signs are slight, and, in addition, there is not and has not been much rise in temperature, if the pulse is not fast and the general constitutional symptoms are not severe, it will probably be quite safe to wait till the attack has passed off before performing the operation. If, however, the pulse is very fast and remains so, and the constitutional symptoms are severe, even though there is no sign of swelling over the appendix and not very marked tenderness, immediate operation is, I believe, the safest procedure. If there are signs of abscess, operation is, of course, indicated at once. No one would now think of leaving an abscess unopened, either of the appendix or anywhere else. Most surgeons in this country agree that the immediate operation is the safest treatment for appendicitis in children, except in those cases where there is a reasonable certainty that a gangrenous appendix or abscess does not exist. And it is better to err on the side of operation at once than to leave a gangrenous appendix or an abscess to burst into the peritoneal cavity. Children, as a rule, recover rapidly if operated upon before general infection has occurred; but the prognosis is most grave after such infection is once established.

I cannot remember ever having seen a case in which serious harm resulted from immediate operation in a case of appendicitis in a child, but I have a most vivid recollection of several cases where a child's life was lost through delaying the operation. I remember one case in particular in which the operation was performed in spite of the medical opinion being in favour of delay, and the child's life was certainly saved. The patient was a little girl of seven. She had had a sharp attack of appendicitis, and had been ill for two or three days. The doctor thought it advisable to send for the surgeon, but in the meantime the child quite suddenly got very much better. When we saw her the pain had disappeared, the temperature had come down a great deal, and the child was quite cheerful, and said she was much better. We arrived, in fact, to find everybody congratulating themselves that, after all, the operation would be unnecessary. On examining the child, however, there was still some tenderness over the appendix region, and the pulse was still very fast. It was therefore considered safer to perform an immediate operation. On opening the abdomen a large abscess containing four or five ounces of pus was discovered, which appeared to be on the very point of bursting into the peritoneal cavity. This was drained, and the child made a good recovery. What had probably happened was that the walls of the abscess had commenced to stretch and give way, and the tension had thus been relieved; this had temporarily stopped the pain, and by diminishing the absorption of toxins had brought down the temperature. Delay in this case would almost certainly have proved fatal.

### The Operation Described.

The most important factor in operations upon children is that of time. Children *will not* stand long operations, and it is of the utmost importance to operate as rapidly as possible and to do as little as possible. For this reason if we are dealing with an acute appendix abscess we should be content simply to open and drain it, and no attempt should be made to remove the appendix unless it can be got out quite easily and without unduly lengthening the operation. It is much easier to do too much than too little when operating on children.

The greatest care should be taken to keep the child warm during the operation and not to expose the abdomen to cold more than can possibly be helped. Personally, I prefer ether or C.E. mixture to chloroform for the anæsthetic, as it has a less depressing effect upon the central nervous system. The incision should whenever possible be made so that the muscles can be separated and not cut across. If the muscles are cut across there is considerable danger of ventral hernia, as the incision, as compared to the size of the child, is often necessarily a large one. A very good plan in applying the dressings is to put two broad pieces of strapping round the body so as to hold the dressing at the top and bottom; besides preventing the dressings from shifting, it has the additional advantage of supporting the abdominal wound and preventing the stitches from cutting through, while at the same time it does away with the necessity of a light binder, which is very liable to embarrass the respiration.

If the appendix has not been removed at the operation it is always advisable to warn the parents that a second operation may be necessary later on In many cases when an abscess has formed and been opened the appendix gives no further trouble, but this cannot be depended upon. Fresh attacks of appendicitis may occur, and if the parents have not been warned of this they not infrequently think that the operation, which was not done to remove the appendix, but to save the child's life, was badly done. I recently operated upon a girl who had an acute appendix abscess opened, by which I have no doubt that her life was saved. Unfortunately her parents were led to suppose that there would be no further trouble after the wound had healed. The child, however, had five subsequent attacks of appendicitis, and I removed a very much diseased appendix which had a tight stricture at the site of the earlier perforation. When general peritonitis has already occurred the results of operation are very bad and only a very few cases recover. An operation should, however, always be done, as a certain number of cases do recover after operation, while without operation recovery is almost impossible. In such circumstances the operation should consist of free incisions and the establishment of good drainage both of the appendix area and the pelvic pouch. It is particularly important in these cases to make the operative procedure as brief as possible.

### After-Treatment.

Careful after-treatment is always of great importance and is, I believe, by no means the least important part of the treatment of any operation case. The after-treatment is specially important in children, as on it, recovery in bad cases often depends. If there is any shock present after the operation the foot of the cot should be well raised and a warm water enema given. The child should be well covered up and kept warm, but a mistake which nurses frequently make, and which one should always look out for, is to allow heavy bedclothes to rest upon the child's chest. Free respiration is of the greatest importance to children, and in children the respiration is mainly abdominal. Abdominal breathing is seriously interfered with by the weight of bedclothes. All weight should be entirely taken off the thorax by a cradle, and there should be nothing but a light flannel nightgown over the chest.

Children stand starvation very badly, and it is therefore necessary to begin feeding them as soon as possible after the operation. I give albumen water made from eggs, and sugar, both of which are very good diets. The bowels should be opened as soon as possible by a dose of castor oil, given 24 hours after the operation, or in some cases earlier than this. Enemas are also useful. If the bowels do not act at once an oil or turpentine enema should be given, and the castor oil repeated if necessary. In bad cases, especially when there has been general peritonitis, everything depends on getting the bowels open, and energetic measures in this direction are often necessary.

Children should not be fastened down in bed or kept in one position after the operation. They do much better if allowed to move about within reason. a child, if left alone, will quickly try and get into the most comfortable position, and that of necessity will be the position of greatest rest, and therefore the best. Nature is the best guide in this matter, and a child, when seriously ill, will not try to sit up, and if its little movements are restricted will only fret and tire itself out in trying to move; while if it is helped into the position wanted will often fall asleep at once. Stimulants, and especially strychnine, must be used with the greatest caution in children, and in most cases are best avoided.

